# Advance Care Planning Motivators Among Adults With Serious Illness

**DOI:** 10.1001/jamanetworkopen.2025.41401

**Published:** 2025-11-04

**Authors:** Sarah Nouri, Carine Davila, Stephanie H. Chan, Brian Feltz, Anna Gosline, Rebecca L. Sudore, Christine S. Ritchie

**Affiliations:** 1 Division of Palliative Medicine, Department of Medicine, University of California San Francisco; 2 Division of Palliative Care and Geriatric Medicine, Department of Medicine, Massachusetts General Hospital and Harvard Medical School, Boston; 3 Center for Aging and Serious Illness, Mongan Institute, Massachusetts General Massachusetts Hospital, Boston; 4 Massachusetts Coalition for Serious Illness Care, Boston; 5 Blue Cross Blue Shield of Massachusetts, Boston; 6 3D Research Partners LLC, Harvard, Massachusetts; 7 Flowetik, Boston, Massachusetts; 8 Division of Geriatrics, Department of Medicine, University of California, San Francisco

## Abstract

**Importance:**

Advance care planning (ACP) engagement is poorly understood among people with serious illness.

**Objective:**

Determine whether diagnosis of and worries about serious illness are associated with ACP engagement.

**Design, Setting, and Participants:**

This cross-sectional study was based on a nationally representative survey administered April to May 2021 online or by telephone. Eligible participants were adults and English or Spanish language. Data were analyzed May 2023 to February 2025).

**Exposure:**

Having a serious illness.

**Main Outcomes and Measures:**

Three domains of ACP engagement: discussion with people close to participants, discussion with clinicians, and documentation in writing. Barriers to ACP documentation and worries about serious illness were also assessed. Survey responses were weighted to reflect the US Census population. χ^2^ tests and multivariable logistic regression models were adjusted for covariables to assess associations between serious illness and each ACP outcome.

**Results:**

Among 1854 participants (468 [weighted percentage, 25.2%] aged 65 years or older; 955 women [weighted percentage, 51.9%]; 193 Black [weighted percentage, 12.0%], 288 Hispanic [weighted percentage, 16.7%], 1218 White [weighted percentage, 61.2%]), 367 (weighted percentage, 20%) had serious illness. Overall, 1254 (weighted percentage, 65.9%) had engaged in ACP (283 of 367 [weighted percentage, 76.5%] serious illness vs 971 of 1487 [weighted percentage, 62.6%] without serious illness; *P* < .001); 1071 (weighted percentage, 55.3%) discussed ACP with people close to them, 462 (weighted percentage, 22.5%) discussed with clinicians, and 650 (weighted percentage, 32.8%) documented a surrogate. People with serious illness had higher odds than those without having discussed choices for surrogates and medical wishes with people close to them (aOR, 1.57 [95% CI, 1.19-2.07] and 1.66 [95% CI, 1.26-2.19]) and with clinicians (aOR, 2.16 [95% CI, 1.63-2.88] and 2.22 [95% CI, 1.67-2.94]). Serious illness status was not associated with ACP documentation. The most common barriers to documentation were not having thought about it (489 of 1171 [weighted percentage, 43.1%]) and assuming surrogates would know what they want (369 of 1171 [weighted percentage, 32.2%]). Participants who were worried (vs not worried) about being able to afford care, surrogates making the best or right decisions, having access to the best treatments, or having high stress or symptoms had significantly higher rates of ACP engagement.

**Conclusions and Relevance:**

In this survey study of adults in the US, we found that ACP discussions with someone close to them were high, yet discussions with clinicians and documentation were low, even among people with serious illness. Our findings offer insights into how people’s worries about serious illness care may affect their engagement in ACP, and how to leverage these insights to tailor patient-facing messaging about ACP.

## Key Points

**Question:**

Are serious illness diagnosis and worries about serious illness associated with advance care planning (ACP) engagement?

**Findings:**

In this cross-sectional study including a nationally representative survey of 1854 participants, those with serious illness were significantly more likely than those without to have discussed ACP with people close to them and clinicians. Participants who were worried (vs not worried) about surrogates making the best or right decisions or having access to best treatments had significantly higher ACP engagement.

**Meaning:**

These findings suggest that framing ACP as a means of helping patients and their surrogates make the best medical decisions may be effective in increasing ACP engagement.

## Teaser

This cross-sectional study of nationally representative survey of US adults compares prevalence of advance care planning among those with vs without serious illness.

## Introduction

Advance care planning (ACP), or the process by which people and their surrogate decision-makers prepare for communication and medical decision-making, is associated with higher satisfaction with communication and care and lower surrogate distress.^1^ Recent discussions about the definition of ACP have led to a renewed conceptual framework that includes both in-the-moment and future decision-making across the life course.^2^ Given the complexity of decision-making related to serious illnesses, the relative need to explore goals for medical care is higher for people with serious illness compared to healthy individuals.^2,3,4^ Yet, research exploring how people with serious illness engage in ACP is limited.

Prior research has demonstrated that overall rates of ACP engagement are low, particularly among populations experiencing systemic disadvantage, such as racially or ethnically minoritized populations and those with lower socioeconomic status.^5,6,7,8,9,10,11,12,13^ A significant body of research has explored barriers to and facilitators of ACP and designed interventions to increase ACP engagement.^14,15,16,17,18,19,20,21^ Yet, ACP documentation in the US has not meaningfully increased over recent years, even since the coronavirus pandemic resulted in unprecedented levels of death and disability and persistent public messaging urged people to document ACP.^10,22,23,24,25^

Therefore, to meaningfully improve communication and medical decision-making, it is necessary to more deeply and more widely explore ACP experiences, particularly among people with serious illness, for whom ACP engagement may be more urgent. Our prior work suggested that health care–related worries may influence ACP engagement, but there is little understanding of how specific worries around serious illness diagnosis may influence ACP engagement.^26^ In this cross-sectional study, we examine ACP engagement, ACP barriers, and worries related to serious illness in a large, nationally representative sample of adults with and without serious illness to gain new insights into improving ACP and other communication interventions for people with serious illness. We hypothesized that serious illness diagnosis was associated with higher ACP engagement and that exploring worries about serious illness may shed light on facilitators of ACP engagement.

## Methods

### Study Setting and Participants

The Massachusetts Coalition for Serious Illness Care (MCSIC) designed and administered a cross-sectional survey from April to May 2021 to assess people’s perceptions of health care, including advance care planning.^27,28,29^ They administered surveys in English or Spanish and online or by telephone. The NORC (National Opinion Research Center) at the University of Chicago recruited a nationally representative sample of adults aged 18 years or older in the US via a probability based panel (AmeriSpeak) and obtained written informed consent from all participants.^30^ Black and Hispanic individuals, people with household income below $50 000 per year, people with serious illness, people with self-reported disability, and older adults (ie, older than 65 years) were oversampled. The Harvard Longwood Campus and University of California, San Francisco institutional review boards approved this study. We followed best practices from American Association for Public Opinion Research (AAPOR) reporting guideline for survey research.

### ACP Engagement Outcomes

The survey assessed 3 domains of ACP engagement, within which we examined 7 dichotomous (yes, no) outcome measures among participants: discussions with someone close to them (about their choice for a surrogate decision-maker or their medical wishes); discussions with clinicians (about their choice for a surrogate decision-maker, their medical wishes, or a desire to discuss medical wishes with a clinician among those who had not done so); and documentation in writing (about their choice for a surrogate decision-maker or their medical wishes) (eTable 1 in Supplement 1). We also examined a dichotomous overall ACP engagement outcome, which we defined as whether a participant responded “yes” to having had discussions with someone close to them or with clinicians about or having documented in writing their choice for a surrogate decision-maker or their medical wishes vs “no” to all.

### Primary Independent Variable and Covariables

The primary independent variable was having a serious illness (yes, no), which was defined as having both (1) a self-reported diagnosis of diabetes, asthma, lung disease, heart disease, cancer, dementia, chronic kidney disease, depression, anxiety, or other serious mental illness, and (2) a self-reported decline in health over the prior 12 months.

Self-reported covariables obtained from the survey were: age (grouped as 18 to 44 years, 45 to 64 years, 65 to 74 years, 75 years or older), gender (man, woman, transgender, nonbinary), race or ethnicity (Hispanic, non-Hispanic Asian, non-Hispanic Black, non-Hispanic other, non-Hispanic White), household income (below $30 000, $30 000 to under $60 000, $60 000 to under $100 000, $100 000 or higher), living in a metropolitan area (yes, no), region in the US (Northeast, Midwest, South, West), marital status (married or living with a partner, previously married, never married), importance of faith or spirituality (very, somewhat, not too important, not at all), and self-efficacy measured as confidence managing health problems (no health problems, not very, somewhat, very).

### Exploratory Outcomes: Barriers to ACP Documentation and Worries About Serious Illness

Among those who reported not having documented their medical wishes in writing, the survey included a close-ended question asking about reasons for not having done so. Barriers to discussions with people close to them and with clinicians were not assessed. All participants were asked about the following worries in the setting of current or future serious illness: affording care, surrogates making the best or right decisions for them, access to best treatments, high stress or symptom burden, caregiver burden, making medical decisions, managing medical tasks, discrimination, and independence or function.

### Statistical Analysis

All analyses applied survey weights to reflect the US Census population and were performed using IBM SPSS Statistics version 29 (IBM) from May 2023 to February 2025. We performed descriptive statistics on dependent and independent variables. For each outcome variable, we conducted χ^2^ tests and 3 multivariable logistic regression models to examine outcomes’ associations with serious illness: (1) unadjusted, (2) partially adjusted for demographic characteristics (age, gender, race and ethnicity, income, metropolitan area, region), (3) fully adjusted for demographic characteristics and other covariables known from prior research to be associated with ACP (marital status, importance of faith or spirituality, and self-efficacy).^31,32,33^ We used χ^2^ tests to determine whether ACP-related barriers and worries were associated with serious illness, and whether worries were associated with ACP engagement outcomes. We used a significance threshold of *P* < .05 in 2-sided tests.

## Results

### Participant Characteristics

The survey completion rate was 30.3% (margin of error was +3.1%). Of the 1854 total participants, 367 (weighted percentage, 19.8%) had serious illness, 468 (weighted percentage, 21.7%) were aged 65 years or older, and 955 (weighted percentage, 51.9%) identified as female; 288 participants (weighted percentage, 16.7%) identified as Hispanic, 193 (weighted percentage, 12.0%) as Black, and 50 (weighted percentage, 6.5%) as Asian (Table 1). Compared with those without serious illness, participants with serious illness were more likely to be women (221 of 367 [weighted percentage, 64.6%]; *P* < .001), have lower income (247 of 367 [weighted percentage, 58.8%] under $60 000 household income; *P* < .001), or live in the South (131 of 367 [weighted percentage, 42.2%]; *P* = .03), and were less likely to live in a metropolitan area (297 of 367 [weighted percentage, 81.5%]; *P* = .02) or have confidence managing their health problems (66 of 367 [weighted percentage, 18.4%]; *P* < .001).

**Table 1.** Participant Characteristics

Characteristic	Participants, No. (weighted %)^a^			*P* value
	Overall (N = 1854)	Serious illness (n = 367)	No serious illness (n = 1487)	
Age, y				
18-44	777 (46.8)	139 (43.2)	638 (47.6)	.06
45-64	609 (31.6)	114 (29.8)	495 (32.0)	
65-74	325 (14.9)	73 (17.7)	252 (14.2)	
≥75	143 (6.8)	41 (9.4)	102 (6.2)	
Gender^b^				
Man	867 (46.2)	135 (33.7)	732 (49.1)	<.001
Woman	955 (51.9)	221 (64.6)	734 (49.0)	
Transgender or nonbinary	22 (1.0)	9 (1.6)	13 (0.9)	
Race and ethnicity^c^				
Asian, non-Hispanic	50 (6.5)	9 (3.9)	41 (7.1)	.08
Black, non-Hispanic	193 (12.0)	35 (12.5)	158 (11.8)	
Hispanic	288 (16.7)	55 (16.4)	233 (16.7)	
White, non-Hispanic	1218 (61.2)	238 (61.9)	980 (61.1)	
Other, non-Hispanic	105 (3.7)	30 (54)	75 (3.3)	
Household income, $				
<30 000	437 (17.8)	133 (27.3)	304 (15.7)	<.001
30 000 to <60 000	526 (25.1)	114 (31.5)	412 (23.7)	
60 000 to <100 000	464 (30.1)	75 (27.9)	389 (30.6)	
≥100 000	427 (26.9)	45 (13.3)	382 (30.0)	
Metropolitan area	1569 (85.4)	297 (81.5)	1272 (86.3)	.02
Region				
Northeast	256 (17.3)	37 (12.0)	219 (18.5)	.03
Midwest	491 (20.7)	101 (21.0)	390 (20.7)	
South	628 (38.0)	131 (42.2)	497 (37.1)	
West	479 (23.9)	98 (24.9)	381 (23.7)	
Marital status				
Married or living with partner	1095 (62)	204 (61.9)	891 (62)	>.99
Previously married	368 (16.6)	89 (17)	279 (16.5)	
Never married	391 (21.5)	74 (21.1)	317 (21.5)	
Importance of faith or spirituality^b^				
Very important	773 (41.2)	149 (44.0)	624 (40.6)	.20
Somewhat important	445 (25.7)	88 (22.9)	357 (26.4)	
Not too important	249 (14.0)	56 (15.1)	193 (13.8)	
Not important at all	341 (16.8)	69 (17.1)	272 (16.7)	
Confidence managing health problems^b^				
I do not have any health problems	132 (7.2)	4 (0.7)	128 (8.7)	<.001
Not very confident	185 (11.4)	81 (21.1)	104 (9.2)	
Somewhat confident	979 (52.5)	216 (59.7)	763 (50.9)	
Very confident	557 (28.8)	66 (18.4)	491 (31.1)	

^a^ Analyses applied survey weights to reflect the US Census population.

^b^ Missing data: importance of faith or spirituality, less than 3%; gender and confidence managing health problems, less than 1%.

^c^ Survey response options for race and ethnicity included only the categories listed in this table (non-Hispanic other was not further divided into additional categories).

### ACP Engagement

Table 2 includes results of unadjusted and fully adjusted analyses of ACP engagement; complete multivariable model results are in eTables 2 through 9 in Supplement 1. Overall, 1254 of 1854 participants (weighted percentage, 65.9%) had engaged in some form of ACP. Specifically, 55% discussed ACP with people close to them (1052 [weighted percentage, 54.3%] discussed surrogate, 1071 [weighted percentage, 55.3%] discussed medical wishes), 440 participants (weighted percentage, 22.2%) discussed surrogate or medical wishes with clinicians, and 31% documented ACP in writing (650 [weighted percentage, 32.8%] documented surrogate and 587 [weighted percentage, 29.9%] documented medical wishes). Overall, any form of ACP engagement was higher among those with serious illness compared with those without serious illness (unadjusted, 283 of 367 participants [weighted percentage, 76.5%] vs 971 of 1487 participants [weighted percentage, 62.6%]; *P* < .001; fully adjusted aOR, 1.91 [95% CI, 1.41-2.58]).

**Table 2.** Advance Care Planning (ACP) Outcomes by Self-Reported Serious Illness, Including Unadjusted and Adjusted Analyses

Outcome	Unadjusted analyses				Multivariable logistic regression, aOR (95% CI)^a^^,^^b^
	Participants, No. (weighted %)^c^			*P* value	
	Overall (N = 1854)	Serious illness (n = 367)	No serious illness (n = 1487)		
Engagement in any form of ACP below	1254 (65.9)	283 (76.5)	971 (62.6)	<.001	1.91 (1.41-2.58)
Close relation					
Discussed surrogate	1052 (54.3)	248 (62.4)	804 (52.5)	.001	1.57 (1.19-2.07)
Discussed medical wishes with someone close to them	1071 (55.3)	243 (63.2)	828 (53.5)	.004	1.66 (1.26-2.19)
Clinician					
Discussed surrogate	440 (22.2)	130 (33.7)	310 (19.5)	<.001	2.16 (1.63-2.88)
Discussed medical wishes	462 (22.5)	133 (34.8)	329 (19.7)	<.001	2.22 (1.67-2.94)
Wants to talk about medical wishes^a^	697 (46.2)	122 (51.4)	575 (45.2)	0.20	1.46 (1.05-2.01)
Already documented					
Surrogate	650 (32.8)	140 (36.2)	510 (32.0)	0.40	1.12 (0.84-1.50)
Medical wishes	587 (29.9)	134 (35.3)	453 (28.7)	.07	1.34 (0.99-1.80)

^a^ Participants with missing predictor data (<3%) were excluded from multivariable analysis. A total of 1381 participants answered positively for wanting to talk to a clinician about medical wishes, because this was only asked of the subset of participants who reported not having talked to a clinician.

^b^ Fully adjusted models were adjusted for age, gender, race and ethnicity, income, metropolitan area, region, marital status, importance of faith or spirituality, and self-efficacy. Complete model results are available in eTables 2 through 9 in Supplement 1.

^c^ Analyses applied survey weights to reflect the US Census population.

For discussions with someone close to them, people with serious illness were more likely than those without serious illness to have discussed their choices for surrogate decision-makers (248 of 367 [weighted percentage, 62.4%] vs 804 of 1487 [weighted percentage, 52.5%]; *P* = .001) and medical wishes (243 of 367 [weighted percentage, 63.2%] vs 828 of 1487 [weighted percentage, 53.5%]; *P* = .004). These differences persisted in fully adjusted analyses (surrogate: aOR, 1.57 [95% CI, 1.19-2.07]; medical wishes: aOR, 1.66 [95% CI, 1.26-2.19]).

For discussions with clinicians, people with serious illness were also more likely than those without serious illness to have discussed their choices for surrogate decision-makers (133 of 367 [weighted percentage, 34.8%] vs 329 of 1487 [weighted percentage, 19.7%]; *P* < .001) and medical wishes (130 of 367 [weighted percentage, 33.7%] vs 310 of 1487 [weighted percentage, 19.5%]; *P* < .001). These differences persisted in fully adjusted analyses (surrogate: aOR, 2.16 [95% CI, 1.63-2.88]; medical wishes: aOR, 2.22 [95% CI, 1.67-2.94]). Among those who had not discussed ACP with their clinicians (1381 participants), in fully adjusted analysis, people with serious illness had higher odds of wanting to discuss medical wishes with their clinicians compared with those without serious illness (aOR, 1.46 [95% CI, 1.05-2.01]).

For documentation of a surrogate decision-maker or medical wishes, there were no significant differences in unadjusted or fully adjusted analyses between those with vs without serious illness in documentation.

### Barriers to ACP Documentation

**Figure 1.**
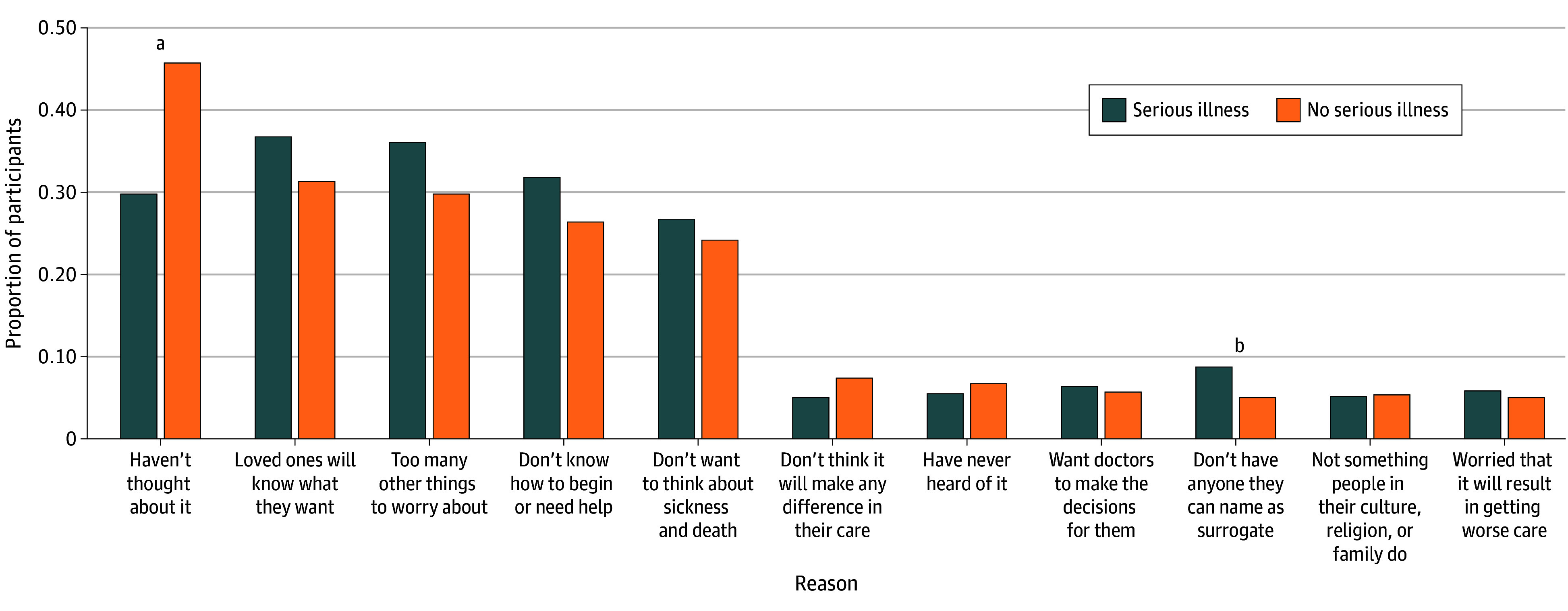
Frequency of Reasons Why Participants Have Not Written Down Their Wishes for Their Medical Care, by Self-Reported Serious Illness ^a^*P* < .001. ^b^*P* = .03.

Among those who had not documented their medical wishes in writing (1171 participants), the most commonly listed reasons for not having done so were not having thought about it because they were too young or healthy (489 [weighted percentage, 43.1%]), feeling people close to them would know what they want (369 [weighted percentage, 32.2%]), having too many other things to worry about (357 [weighted percentage, 30.7%]), not knowing how to begin (306 [weighted percentage, 27.3%]), and not wanting to think about sickness and death (294 [weighted percentage, 24.7%]) (Figure 1; eTable 10 in Supplement 1). A small proportion of participants reported thinking these documents would not make any difference in their care (84 [weighted percentage, 6.8%]), never having heard of ACP (66 [weighted percentage, 6.5%]), wanting their doctors to make decisions for them (76 [weighted percentage, 5.7%]), not having anyone they could count on as a surrogate (81 [weighted percentage, 5.6%]), ACP not being something people in their culture, religion, or family do (40 [weighted percentage, 5.4%]), and concern that having documented ACP will mean they get worse care (53 [weighted percentage, 5.1%]). For people with serious illness compared with those without serious illness, reasons were proportionally similar except a lower proportion (56 of 213 [weighted percentage, 29.8%]) said they had not thought about it because they were too young or healthy, and a higher proportion (26 of 213 [weighted percentage, 8.8%]) said they did not have anyone they could count on as a surrogate decision-maker.

### Worries About Current or Future Serious Illness

**Figure 2.**
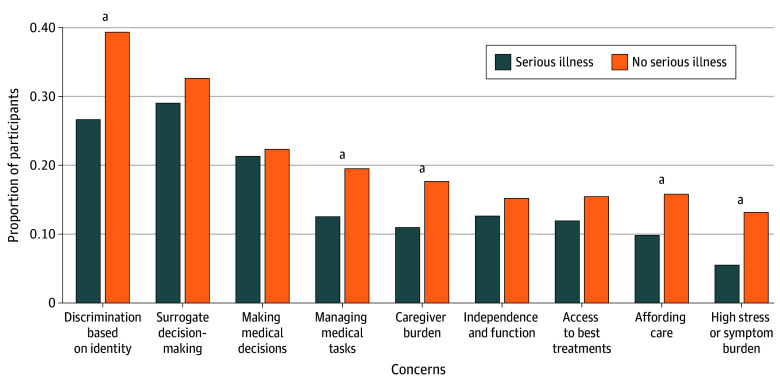
Frequency of Participants’ Worries in the Setting of Serious Illness, by Self-Reported Serious Illness ^a^*P* < .05.

**Figure 3.**
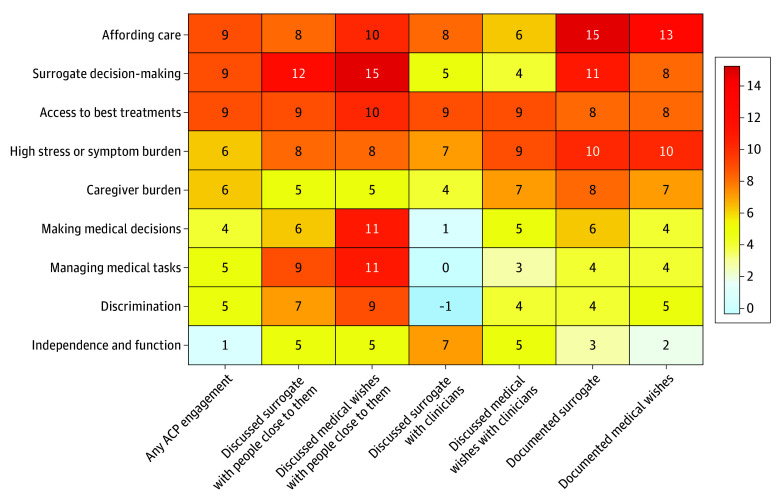
Heatmap Showing the Difference in Advance Care Planning (ACP) Engagement Between People Who Are Worried vs Not Worried Across Different Concerns Rows represent specific worries; columns represent types of ACP engagement. The numbers in the table are the differences in percentage of ACP engagement between people who are worried vs not worried (eg, 9 in the first cell means people who were worried about affording care were 9% more likely to have any ACP engagement compared to people who were not worried about affording care). Numbers in bold represent a statistically significant difference (*P* < .05).

Compared with people without serious illness, people with serious illness were less likely to be worried about discrimination, managing medical tasks, caregiver burden, having trouble affording care, or having high stress or symptom burden (Figure 2; eTable 10 in Supplement 1). Overall, people who had any worries, regardless of serious illness diagnosis, were more likely to engage in ACP (Figure 3; eTable 11 in Supplement 1). Worries about having trouble affording care, surrogates making the best or right decisions, and having access to the best treatment options were associated with the greatest differences in ACP engagement (approximately 9% difference). This was followed by worries about having high stress or symptom burden and about caregiver burden (6% difference). Worries about independence or function were least likely to be associated with differences in ACP engagement.

## Discussion

To the best of our knowledge, this is the first study to use a nationally representative sample to examine ACP engagement, barriers, and motivators in adults with vs without serious illness. We found that over half of people overall and nearly two-thirds of people with serious illness have had discussions with people close to them about their choices for surrogate decision-makers and medical wishes—a higher proportion than that found in research from prior years, suggesting these discussions may be increasing over time.^34^ ACP discussions with clinicians and documentation remain low, but are modestly higher for those with serious illness compared with those without serious illness.^25,32,35^

While the high proportion of people engaging in ACP discussions with people close to them is promising, over a third of people with serious illness reported not having had these discussions. Many participants in this study expressed worry that their surrogate decision-makers would not make the best or right decisions about their care and that they themselves may not understand how to make the best choices for treatment in the setting of serious illness—in other words, both patients and surrogates need better preparation for communication and medical decision-making, as well as more discussions with each other.^36,37,38,39^ Interestingly, people who were worried about these issues were significantly more likely than those who were not worried to engage in ACP—specifically having discussions with people close to them about their medical wishes. This finding suggests that messaging ACP as a means of helping facilitate communication and medical decision-making for patients and their surrogates may be particularly effective in increasing ACP engagement.^40^

In prior work, we developed 5 ACP personas based on engagement and experiences with health care.^26^ Among these personas are “worried action takers” and “disengaged worriers.” The former tend to have high trust and regard for the health care system and significant worries about a future serious illness. The latter tend to have poorer health and self-efficacy and have seen the wishes of people close to them not honored by the health care system. In this study, we found that certain worries (eg, being able to afford care, surrogates making the best or right decisions, caregivers’ experiencing burden) were associated with significantly higher ACP engagement rates. Conversely, other worries (eg, not being able to continue living in one’s preferred place) were not significantly associated with ACP engagement. This information provides valuable insight into ACP behaviors and into messaging that may or may not motivate people to engage in ACP. Together with our findings, it is possible that further exploring these personas and understanding how worries about serious illness connect to the perceived value of ACP behaviors may help design more effective interventions that will fully engage people with serious illness and their surrogate decision-makers and thereby improve communication and decision-making.

While it is encouraging that rates of ACP conversations are higher in this study compared with prior research, our findings confirm reports that ACP documentation remains low, as documented in prior studies.^22,34,35^ This study also adds to the existing literature by exploring a wide array of potential reasons for this lack of documentation increase. At the start of the coronavirus pandemic, many clinicians and ACP researchers expected and advocated for higher patient engagement in ACP given the unprecedented morbidity and mortality in our recent history.^23^ Yet, data from this study, which were collected well into the pandemic in 2021, demonstrate rates of ACP documentation similar to those prior to the pandemic.^22^ Barriers to documentation were similar to those noted in prior research, including a lack of awareness or knowledge about ACP, a desire to avoid thinking about sickness and death, competing priorities, and an assumption that people close to them would know what participants would want.^35,41,42^ While ACP engagement takes several forms, documentation remains an important component of ACP as a legally recognized communication of patients’ designated surrogate decision-makers or medical wishes. For example, a POLST form with a do not resuscitate order may be the first form of communication an emergency response provider can rely on to honor a patient’s wishes, or an advance directive may be the only form of communication designating the surrogate decision-maker for a hospitalized patient who cannot otherwise communicate.^6,43,44^ By walking patients step-by-step through various scenarios and decisions, the exercise of documentation itself can also be a tool for clarifying values and goals and encouraging ACP discussions.

Importantly, ACP discussions and documentation are but one tool to honor patients’ medical preferences within complex health care systems. Although ACP aims to prepare people for communication and medical decision-making, it cannot mitigate, on its own, the challenges people face in navigating health care systems, communicating effectively with clinicians who have limited time and training in communication, or participating in shared decision-making when facing relational power and informational discrepancies, nor can it mitigate ongoing structural racism and discrimination. Notably, over a third of participants expressed worry that they would not get the best care because of their identity, suggesting that discrimination may play a significant role in lack of ACP engagement. The concern that formal documentation of patients’ wishes would result in worse care has been echoed in prior studies of marginalized populations due to racism and lack of trustworthiness of health care systems.^32,41,42^ It is possible that ACP documentation is perceived publicly as permission to restrict care (eg, a do-not-resuscitate order) rather than a means of documenting values and surrogates—particularly given the default culture in US health care of high-intensity, life-sustaining care—thereby raising concerns that ACP documentation may compound disparities in management of chronic or serious illnesses among certain marginalized populations.^45,46^ Furthermore, many people in this study focused on financial concerns, which are outside of the purview of standard ACP in medical settings. Advances in ACP, including a focus on conversations, improved clinician documentation, and electronic health record reform (eg, interoperability) must be combined with advances in health care reform to center equity, access to palliative care, and ongoing clinician training in communication.^2,44,47,48^

### Strengths and Limitations

The strength of this study was the nationally representative sample. Limitations include the exploration of barriers being focused only on ACP documentation rather than also on ACP discussions. Understanding barriers to discussions with surrogates is a critical next step in research, since a tremendous body of research has demonstrated the value of ACP in reducing surrogate decision-makers’ distress. We were unable to fully assess the intersectionality between serious illness and sociodemographic factors known to be associated with low engagement in ACP due to small sample sizes. Nevertheless, we were successfully able to recruit a uniquely diverse group of participants. Data were collected approximately one year into the coronavirus pandemic; this proximity in timing may have contributed to the high rates of ACP discussions with people close to participants compared to prepandemic studies, although notably rates of discussions with clinicians and documentation were similar to those described in prepandemic studies.

## Conclusions

This cross-sectional survey study of adults in the US demonstrated that ACP discussions with people close to patients were high, while ACP documentation and discussions with clinicians were low. Our findings suggest that further work is needed to understand and address barriers at the clinician (eg, patient-clinician rapport, clinician readiness and comfort in discussing ACP) and health system (eg, discrimination, lack of trustworthiness) levels. These findings also highlight areas in which both ACP and serious illness care must grow to address people’s concerns, and how harnessing messaging that speaks to and alleviates worries about serious illness communication and medical decision-making may motivate greater engagement in ACP. Next steps must include a greater focus on motivators and facilitators of ACP discussions with surrogates as well as clinicians and documentation, as well as on exploring how ACP can be redefined and implemented within health systems to address the worries that people have when facing serious illness.

## Notes

Supplement 1. **eTable 1.** Outcome Measures **eTable 2.** Odds of Having Engaged in Any Form of Advance Care Planning (Discussion With Someone Close to Them, Discussion With Clinicians, and/or Documentation), by Self-Reported Serious Illness **eTable 3.** Odds of Having Discussed Choice for a Surrogate Decision-Maker With Someone Close to Them, by Self-Reported Serious Illness **eTable 4.** Odds of Having Discussed Medical Wishes With Someone Close to Them, by Self-Reported Serious Illness **eTable 5.** Odds of Having Discussed Choice for Surrogate Decision-Maker With Clinicians, by Self-Reported Serious Illness **eTable 6.** Odds of Having Discussed Medical Wishes With Clinicians, by Self-Reported Serious Illness **eTable 7.** Odds of Wanting to Discuss Medical Wishes With Clinicians Among Participants Who Have Not Previously Discussed, by Self-Reported Serious Illness **eTable 8.** Odds of Having Documented Choice for Surrogate Decision-Maker in Writing, by Self-Reported Serious Illness **eTable 9.** Odds of Having Documented Medical Wishes in Writing, by Self-Reported Serious Illness **eTable 10.** Frequency of ACP-Related Barriers and Worries by Self-Reported Serious Illness **eTable 11.** Engagement in ACP Among People Who Are Worried vs Not Worried

Supplement 2.
Data Sharing Statement
