# Supplementary material for: Advance Care Planning Motivators Among Adults With Serious Illness
Source: JAMA Netw Open. 2025 Nov 4;8(11):e2541401. doi: 10.1001/jamanetworkopen.2025.41401 (PMC12587200; doi:10.1001/jamanetworkopen.2025.41401)
Supplement: Supplement 1. — eTable 1. Outcome Measures eTable 2. Odds of Having Engaged in Any Form of Advance Care Planning (Discussion With Someone Close to Them, Discussion With Clinicians, and/or Documentation), by Self-Reported Serious Illness eTable 3. Odds of Having Discussed Choice for a Surrogate Decision-Maker With Someone Close to Them, by Self-Reported Serious Illness eTable 4. Odds of Having Discussed Medical Wishes With Someone Close to Them, by Self-Reported Serious Illness eTable 5. Odds of Having Discussed Choice for Surrogate Decision-Maker With Clinicians, by Self-Reported Serious Illness eTable 6. Odds of Having Discussed Medical Wishes With Clinicians, by Self-Reported Serious Illness eTable 7. Odds of Wanting to Discuss Medical Wishes With Clinicians Among Participants Who Have Not Previously Discussed, by Self-Reported Serious Illness eTable 8. Odds of Having Documented Choice for Surrogate Decision-Maker in Writing, by Self-Reported Serious Illness eTable 9. Odds of Having Documented Medical Wishes in Writing, by Self-Reported Serious Illness eTable 10. Frequency of ACP-Related Barriers and Worries by Self-Reported Serious Illness eTable 11. Engagement in ACP Among People Who Are Worried vs Not Worried [file jamanetwopen-e2541401-s001.pdf]

Supplemental Online Content

Nouri S, Davila C, Chan SH, et al. Advance care planning motivators among adults with serious illness. *JAMA Netw Open.* 2025;8(11):e2541401.
doi:10.1001/jamanetworkopen.2025.41401

eTable 1. Outcome Measures

eTable 2. Odds of Having Engaged in Any Form of Advance Care Planning (Discussion With Someone Close to Them, Discussion With Clinicians, and/or Documentation), by Self-Reported Serious Illness

eTable 3. Odds of Having Discussed Choice for a Surrogate Decision-Maker With Someone Close to Them, by Self-Reported Serious Illness

eTable 4. Odds of Having Discussed Medical Wishes With Someone Close to Them, by Self-Reported Serious Illness

eTable 5. Odds of Having Discussed Choice for Surrogate Decision-Maker With Clinicians, by Self-Reported Serious Illness

eTable 6. Odds of Having Discussed Medical Wishes With Clinicians, by Self-Reported Serious Illness

eTable 7. Odds of Wanting to Discuss Medical Wishes With Clinicians Among Participants Who Have Not Previously Discussed, by Self-Reported Serious Illness

eTable 8. Odds of Having Documented Choice for Surrogate Decision-Maker in Writing, by Self-Reported Serious Illness

eTable 9. Odds of Having Documented Medical Wishes in Writing, by Self-Reported Serious Illness

eTable 10. Frequency of ACP-Related Barriers and Worries by Self-Reported Serious Illness

eTable 11. Engagement in ACP Among People Who Are Worried vs Not Worried

This supplemental material has been provided by the authors to give readers additional information about their work.

© 2025 Nouri S et al. *JAMA Network Open*.

eTable 1. Outcome measures

Outcome Measures	Survey Question	Answer options	ACP Domain
Discussed choice for surrogate decision-maker with someone close to them	Have you ever had a serious conversation with someone close to you about who will make decisions about your medical care if you can no longer make them on your own?	Yes, no, don't know, do not wish to answer	Discussions with someone close to them
Discussed medical wishes in the setting of serious illness with someone close to them	Have you ever had a serious conversation with someone close to you about your wishes for medical care [if you become seriously ill]?	Yes, no, don't know, do not wish to answer	
Discussed choice for surrogate decision-maker with a clinician	Have you ever had a serious conversation with a health professional about who will make decisions about your medical care if you can no longer make them on your own?	Yes, no, don't know, do not wish to answer	Discussions with clinicians
Discussed medical wishes in the setting of serious illness with a clinician	Have you ever had a serious conversation with a health professional about your wishes for medical care [if you become seriously ill]?	Yes, no, don't know, do not wish to answer	
Wants to talk to a clinician about medical wishes in the setting of serious illness	[Among those who have not talked with their clinicians] Would you want to talk to a health professional about your wishes for medical care [if you become seriously ill]?	Yes, no, don't know, do not wish to answer	
Documented choice for surrogate decision-maker	Do you have a written document that names who you want to make decisions about your medical care if you can no longer make them on your own?	Yes, no, don't know, do not wish to answer	Documentation in writing
Documented medical wishes in the setting of serious illness	Do you have a written document that describes your wishes for medical care [if you become seriously ill]?	Yes, no, don't know, do not wish to answer	
ACP-Related Barriers and Worries	Survey Question	Answer options	
Barriers	Are any of the following a reason you have not written down your wishes for your medical care [if you became seriously ill]? [see Appendix Table J]	Yes, no, don't know, do not wish to answer	
Worries	How worried are you about the following [if you were to become seriously ill]? [see Appendix Table J]	Not at all, a little bit, somewhat, very	

© 2025 Nouri S et al. *JAMA Network Open*.

eTable 2. Odds of having engaged in any form of advance care planning (discussion with someone close to them, discussion with clinicians, and/or documentation), by self-reported serious illness.

	Odds ratio (95% CI); N = 1794		
**Predictor**	**Unadjusted**	**Partially adjusted***	**Fully adjusted^**
Serious illness			
No	Ref	Ref	Ref
Yes	1.86 (1.42, 2.45)	1.88 (1.40, 2.51)	1.91 (1.41, 2.58)
Age			
18-44		Ref	Ref
45-64		1.49 (1.19, 1.88)	1.32 (1.04, 1.69)
65-74		4.53 (3.11, 6.60)	3.84 (2.58, 5.69)
75+		8.16 (4.18, 15.95)	7.49 (3.74, 14.99)
Gender			
Male		Ref	Ref
Female		1.31 (1.06, 1.62)	1.25 (1.01, 1.56)
Transgender or Non-Binary		6.39 (1.66, 24.67)	6.24 (1.59, 24.49)
Race/Ethnicity			
White, non-Hispanic		Ref	Ref
Black, non-Hispanic		0.65 (0.46, 0.90)	0.67 (0.48, 0.95)
Asian, non-Hispanic		0.77 (0.50, 1.19)	0.91 (0.58, 1.43)
Other, non-Hispanic		1.00 (0.57, 1.76)	1.04 (0.59, 1.85)
Hispanic		0.80 (0.60, 1.06)	0.80 (0.60, 1.08)
Income			
$100,000 or more		Ref	Ref
$60,000 to under $100,000		0.73 (0.55, 0.97)	0.74 (0.56, 0.99)
$30,000 to under $60,000		0.70 (0.52, 0.95)	0.76 (0.56, 1.03)
Less than $30,000		0.49 (0.35, 0.68)	0.61 (0.42, 0.85)
Metro			
No		Ref	Ref
Yes		0.89 (0.64, 1.20)	0.96 (0.70, 1.31)
Region			
Northeast		Ref	Ref
Midwest		0.77 (0.55, 1.09)	0.80 (0.56, 1.14)
South		0.95 (0.70, 1.30)	0.93 (0.68, 1.28)
West		0.66 (0.48, 0.93)	0.65 (0.46, 0.91)
Marital status			
Married or living with partner			Ref
Previously married			0.68 (0.50, 0.93)
Never married			0.58 (0.44, 0.76)
Importance of faith or spirituality			
Very important			Ref

Somewhat important			0.81 (0.62, 1.06)
Not too important			1.13 (0.80, 1.59)
Not important at all			0.88 (0.64, 1.20)
Confidence in managing health problems			
I don't have any health problems			Ref
Not very confident			1.18 (0.71, 1.94)
Somewhat confident			1.59 (1.05, 2.41)
Very confident			1.85 (1.20, 2.87)

*Partially adjusted models were adjusted for age, gender, race/ethnicity, income, metro area, region.

^Fully adjusted models were adjusted for age, gender, race/ethnicity, income, metro area, region, marital status, importance of faith or spirituality, and self-efficacy.

© 2025 Nouri S et al. *JAMA Network Open*.

eTable 3. Odds of having discussed choice for a surrogate decision-maker with someone close to them, by self-reported serious illness.

Predictor	Odds ratio (95% CI); N = 1794		
	**Unadjusted**	**Partially adjusted***	**Fully adjusted^**
Serious illness			
No	Ref	Ref	Ref
Yes	1.50 (1.17, 1.91)	1.47 (1.13, 1.92)	1.57 (1.19, 2.07)
Age			
18-44		Ref	Ref
45-64		1.48 (1.18, 1.85)	1.30 (1.02, 1.65)
65-74		4.47 (3.22, 6.23)	3.65 (2.57, 5.19)
75+		4.79 (2.99, 7.68)	4.25 (2.57, 7.01)
Gender			
Male		Ref	Ref
Female		1.39 (1.14, 1.71)	1.32 (1.07, 1.62)
Transgender or Non-Binary		3.57 (1.25, 10.16)	3.55 (1.22, 10.37)
Race/Ethnicity			
White, non-Hispanic		Ref	Ref
Black, non-Hispanic		0.59 (0.43, 0.82)	0.67 (0.47, 0.92)
Asian, non-Hispanic		0.70 (0.46, 1.08)	0.87 (0.56, 1.35)
Other, non-Hispanic		1.17 (0.68, 1.99)	1.23 (0.71, 2.13)
Hispanic		0.94 (0.71, 1.25)	0.96 (0.71, 1.28)
Income			
$100,000 or more		Ref	Ref
$60,000 to under $100,000		0.83 (0.68, 1.23)	0.87 (0.66, 1.14)
$30,000 to under $60,000		0.66 (0.50, 0.88)	0.74 (0.55, 0.99)
Less than $30,000		0.52 (0.37, 0.71)	0.67 (0.48, 0.95)
Metro			
No		Ref	Ref
Yes		0.92 (0.68, 1.23)	0.99 (0.74, 1.34)
Region			
Northeast		Ref	Ref
Midwest		1.04 (0.75, 1.45)	1.08 (0.77, 1.51)
South		1.14 (0.85, 1.53)	1.14 (0.84, 1.53)
West		0.94 (0.69, 1.29)	0.90 (0.66, 1.25)
Marital status			
Married or living with partner			Ref
Previously married			0.62 (0.46, 0.83)
Never married			0.45 (0.34, 0.59)
Importance of faith or spirituality			
Very important			Ref
Somewhat important			0.96 (0.75, 1.24)

© 2025 Nouri S et al. *JAMA Network Open*.

Not too important			1.21 (0.88, 1.68)
Not important at all			1.00 (0.74, 1.35)
Confidence in managing health problems			
I don't have any health problems			Ref
Not very confident			1.04 (0.63, 1.72)
Somewhat confident			1.27 (0.84, 1.93)
Very confident			1.98 (1.28, 3.07)

*Partially adjusted models were adjusted for age, gender, race/ethnicity, income, metro area, region.
^Fully adjusted models were adjusted for age, gender, race/ethnicity, income, metro area, region, marital status, importance of faith or spirituality, and self-efficacy.

© 2025 Nouri S et al. *JAMA Network Open*.

eTable 4. Odds of having discussed medical wishes with someone close to them, by self-reported serious illness.

	Odds ratio (95% CI); N = 1797		
**Predictor**	**Unadjusted**	**Partially adjusted**	**Fully adjusted**
Serious illness			
No	Ref	Ref	Ref
Yes	1.50 (1.14, 1.86)	1.53 (1.17, 1.99)	1.66 (1.26, 2.19)
Age			
18-44		Ref	Ref
45-64		1.54 (1.23, 1.93)	1.38 (1.08, 1.76)
65-74		3.72 (2.70, 5.12)	3.08 (2.18, 4.34)
75+		4.86 (3.00, 7.85)	4.44 (2.66, 7.42)
Gender			
Male		Ref	Ref
Female		1.20 (0.98, 1.47)	1.15 (0.93, 1.42)
Transgender or Non-Binary		5.16 (1.67, 15.95)	5.61 (1.77, 17.83)
Race/Ethnicity			
White, non-Hispanic		Ref	Ref
Black, non-Hispanic		0.59 (0.43, 0.81)	0.66 (0.47, 0.93)
Asian, non-Hispanic		0.94 (0.61, 1.44)	1.22 (0.78, 1.90)
Other, non-Hispanic		0.94 (0.55, 1.61)	0.98 (0.57, 1.70)
Hispanic		0.67 (0.51, 0.89)	0.69 (0.52, 0.93)
Income			
$100,000 or more		Ref	Ref
$60,000 to under $100,000		0.76 (0.58, 0.99)	0.80 (0.61, 1.05)
$30,000 to under $60,000		0.56 (0.42, 0.74)	0.64 (0.47, 0.86)
Less than $30,000		0.46 (0.33, 0.64)	0.61 (0.44, 0.86)
Metro			
No		Ref	Ref
Yes		0.73 (0.54, 0.98)	0.79 (0.58, 1.07)
Region			
Northeast		Ref	Ref
Midwest		0.88 (0.63, 1.23)	0.92 (0.65, 1.28)
South		1.06 (0.79, 1.41)	1.07 (0.79, 1.44)
West		0.83 (0.61, 1.14)	0.82 (0.59, 1.13)
Marital status			
Married or living with partner			Ref
Previously married			0.59 (0.44, 0.80)
Never married			0.49 (0.37, 0.64)
Importance of faith or spirituality			
Very important			Ref
Somewhat important			0.96 (0.74, 1.24)
Not too important			1.27 (0.98, 1.91)
Not important at all			1.06 (0.78, 1.44)

© 2025 Nouri S et al. *JAMA Network Open*.

Confidence in managing health problems			
I don't have any health problems			Ref
Not very confident			0.88 (0.53, 1.47)
Somewhat confident			1.46 (0.96, 2.22)
Very confident			2.30 (1.49, 3.56)

*Partially adjusted models were adjusted for age, gender, race/ethnicity, income, metro area, region.

^Fully adjusted models were adjusted for age, gender, race/ethnicity, income, metro area, region, marital status, importance of faith or spirituality, and self-efficacy.

© 2025 Nouri S et al. *JAMA Network Open*.

eTable 5. Odds of having discussed choice for surrogate decision-maker with clinicians, by self-reported serious illness.

	Odds ratio (95% CI); N = 1790		
**Predictor**	**Unadjusted**	**Partially adjusted**	**Fully adjusted**
Serious illness			
No	Ref	Ref	Ref
Yes	2.13 (1.64, 2.76)	1.97 (1.49, 2.59)	2.16 (1.63, 2.88)
Age			
18-44		Ref	Ref
45-64		1.44 (1.09, 1.90)	1.32 (0.98, 1.78)
65-74		2.07 (1.48, 2.89)	1.69 (1.18, 2.43)
75+		4.20 (2.76, 6.38)	3.49 (2.21, 5.52)
Gender			
Male		Ref	Ref
Female		0.86 (0.68, 1.08)	0.82 (0.64, 1.05)
Transgender or Non-Binary		1.30 (0.43, 3.91)	1.56 (0.51, 4.79)
Race/Ethnicity			
White, non-Hispanic		Ref	Ref
Black, non-Hispanic		1.08 (0.74, 1.59)	1.01 (0.68, 1.51)
Asian, non-Hispanic		1.01 (0.57, 1.78)	1.16 (0.65, 2.08)
Other, non-Hispanic		1.00 (0.53, 1.87)	0.96 (0.51, 1.82)
Hispanic		1.68 (1.22, 3.32)	1.74 (1.25, 2.42)
Income			
$100,000 or more		Ref	Ref
$60,000 to under $100,000		1.18 (0.85, 1.63)	1.17 (0.84, 1.63)
$30,000 to under $60,000		1.44 (1.03, 2.01)	1.45 (1.03, 2.05)
Less than $30,000		1.39 (0.96, 2.02)	1.46 (0.98, 2.17)
Metro			
No		Ref	Ref
Yes		0.83 (0.59, 1.15)	0.85 (0.61, 1.19)
Region			
Northeast		Ref	Ref
Midwest		1.20 (0.82, 1.76)	1.25 (0.85, 1.83)
South		0.96 (0.68, 1.35)	0.97 (0.69, 1.38)
West		0.97 (0.67, 1.41)	0.99 (0.68, 1.44)
Marital status			
Married or living with partner			Ref
Previously married			1.07 (0.77, 1.49)
Never married			0.96 (0.69, 1.35)
Importance of faith or spirituality			
Very important			Ref
Somewhat important			1.04 (0.78, 1.39)
Not too important			0.95 (0.66, 1.38)
Not important at all			0.88 (0.61, 1.27)

© 2025 Nouri S et al. *JAMA Network Open*.

Confidence in managing health problems			
I don't have any health problems			Ref
Not very confident			1.63 (0.74, 3.55)
Somewhat confident			2.31 (1.15, 4.63)
Very confident			4.02 (1.99, 8.10)

*Partially adjusted models were adjusted for age, gender, race/ethnicity, income, metro area, region.
^Fully adjusted models were adjusted for age, gender, race/ethnicity, income, metro area, region, marital status, importance of faith or spirituality, and self-efficacy.

© 2025 Nouri S et al. *JAMA Network Open*.

eTable 6. Odds of having discussed medical wishes with clinicians, by self-reported serious illness.

Predictor	Odds ratio (95% CI); N = 1796		
	**Unadjusted**	**Partially adjusted**	**Fully adjusted**
Serious illness			
No	Ref	Ref	Ref
Yes	2.21 (1.70, 2.85)	2.08 (1.59, 2.73)	2.22 (1.67, 2.94)
Age			
18-44		Ref	Ref
45-64		1.34 (1.02, 1.76)	1.19 (0.89, 1.60)
65-74		1.99 (1.43, 2.77)	1.61 (1.13, 2.30)
75+		3.29 (2.16, 5.02)	2.68 (1.70, 4.23)
Gender			
Male		Ref	Ref
Female		0.91 (0.72, 1.15)	0.88 (0.69, 1.12)
Transgender or Non-Binary		2.97 (1.10, 7.97)	2.96 (1.08, 8.10)
Race/Ethnicity			
White, non-Hispanic		Ref	Ref
Black, non-Hispanic		0.99 (0.67, 1.46)	0.99 (0.66, 1.48)
Asian, non-Hispanic		0.98 (0.56, 1.72)	1.13 (0.64, 2.00)
Other, non-Hispanic		1.44 (0.80, 2.57)	1.40 (0.77, 2.53)
Hispanic		1.45 (1.05, 1.99)	1.48 (1.07, 2.06)
Income			
$100,000 or more		Ref	Ref
$60,000 to under $100,000		0.91 (0.67, 1.25)	0.93 (0.68, 1.28)
$30,000 to under $60,000		0.95 (0.68, 1.32)	0.98 (0.70, 1.38)
Less than $30,000		1.08 (0.75, 1.55)	1.21 (0.82, 1.77)
Metro			
No		Ref	Ref
Yes		0.75 (0.54, 1.04)	0.78 (0.56, 1.09)
Region			
Northeast		Ref	Ref
Midwest		1.19 (0.81, 1.74)	1.23 (0.83, 1.80)
South		1.09 (0.78, 1.53)	1.11 (0.78, 1.57)
West		0.99 (0.68, 1.44)	0.99 (0.68, 1.44)
Marital status			
Married or living with partner			Ref
Previously married			1.01 (0.73, 1.39)
Never married			0.72 (0.51, 1.02)
Importance of faith or spirituality			
Very important			Ref
Somewhat important			0.98 (0.73, 1.31)
Not too important			1.24 (0.87, 1.77)
Not important at all			0.98 (0.69, 1.40)

© 2025 Nouri S et al. *JAMA Network Open*.

Confidence in managing health problems			
I don't have any health problems			Ref
Not very confident			1.69 (0.81, 3.51)
Somewhat confident			1.96 (1.02, 3.76)
Very confident			3.29 (1.71, 6.36)

*Partially adjusted models were adjusted for age, gender, race/ethnicity, income, metro area, region.

^Fully adjusted models were adjusted for age, gender, race/ethnicity, income, metro area, region, marital status, importance of faith or spirituality, and self-efficacy.

© 2025 Nouri S et al. *JAMA Network Open*.

eTable 7. Odds of wanting to discuss medical wishes with clinicians among participants who have not previously discussed, by self-reported serious illness.

Predictor	Odds ratio (95% CI); N = 1334		
	**Unadjusted**	**Partially adjusted**	**Fully adjusted**
Serious illness			
No	Ref	Ref	Ref
Yes	1.24 (0.93, 1.66)	1.29 (0.95, 1.75)	1.46 (1.05, 2.01)
Age			
18-44		Ref	Ref
45-64		1.53 (1.19, 1.96)	1.76 (1.33, 2.32)
65-74		2.78 (1.96, 3.93)	3.11 (2.13, 4.52)
75+		1.44 (0.87, 2.39)	1.54 (0.89, 2.65)
Gender			
Male		Ref	Ref
Female		1.29 (1.03, 1.62)	1.40 (1.10, 1.77)
Transgender or Non-Binary		1.29 (0.36, 4.70)	2.11 (0.56, 8.01)
Race/Ethnicity			
White, non-Hispanic		Ref	Ref
Black, non-Hispanic		0.94 (0.65, 1.35)	0.82 (0.56, 1.21)
Asian, non-Hispanic		1.20 (0.75, 1.92)	1.16 (0.71, 1.89)
Other, non-Hispanic		1.49 (0.80, 2.78)	1.45 (0.77, 2.76)
Hispanic		0.79 (0.58, 1.09)	0.87 (0.63, 1.22)
Income			
$100,000 or more		Ref	Ref
$60,000 to under $100,000		0.55 (0.41, 0.74)	0.52 (0.39, 0.70)
$30,000 to under $60,000		0.67 (0.49, 0.91)	0.66 (0.47, 0.91)
Less than $30,000		0.53 (0.37, 0.75)	0.44 (0.30, 0.64)
Metro			
No		Ref	Ref
Yes		1.04 (0.74, 1.45)	1.00 (0.71, 1.41)
Region			
Northeast		Ref	Ref
Midwest		0.64 (0.45, 0.92)	0.69 (0.48, 1.00)
South		0.64 (0.46, 0.88)	0.67 (0.49, 0.94)
West		0.72 (0.51, 1.02)	0.82 (0.58, 1.18)
Marital status			
Married or living with partner			Ref
Previously married			1.21 (0.86, 1.69)
Never married			2.07 (1.51, 2.83)
Importance of faith or spirituality			
Very important			Ref
Somewhat important			0.83 (0.62, 1.09)
Not too important			0.98 (0.68, 1.42)
Not important at all			0.68 (0.49, 0.96)

© 2025 Nouri S et al. *JAMA Network Open*.

Confidence in managing health problems			
I don't have any health problems			Ref
Not very confident			0.34 (0.19, 0.59)
Somewhat confident			0.80 (0.52, 1.23)
Very confident			0.78 (0.49, 1.24)

*Partially adjusted models were adjusted for age, gender, race/ethnicity, income, metro area, region.
^Fully adjusted models were adjusted for age, gender, race/ethnicity, income, metro area, region, marital status, importance of faith or spirituality, and self-efficacy.

© 2025 Nouri S et al. *JAMA Network Open*.

eTable 8. Odds of having documented choice for surrogate decision-maker in writing, by self-reported serious illness.

	Odds ratio (95% CI); N = 1725		
**Predictor**	**Unadjusted**	**Partially adjusted**	**Fully adjusted**
Serious illness			
No	Ref	Ref	Ref
Yes	1.19 (0.93, 1.53)	1.10 (0.83, 1.46)	1.12 (0.84, 1.50)
Age			
18-44		Ref	Ref
45-64		1.86 (1.44, 2.40)	1.70 (1.30, 2.24)
65-74		5.43 (3.99, 7.40)	4.75 (3.41, 6.63)
75+		13.57 (8.36, 22.01)	12.05 (7.24, 10.06)
Gender			
Male		Ref	Ref
Female		1.32 (1.06, 1.65)	1.26 (0.99, 1.58)
Transgender or Non-Binary		1.91 (0.58, 6.32)	1.82 (0.55, 6.04)
Race/Ethnicity			
White, non-Hispanic		Ref	Ref
Black, non-Hispanic		0.65 (0.44, 0.96)	0.59 (0.40, 0.88)
Asian, non-Hispanic		0.74 (0.44, 1.26)	0.74 (0.43, 1.27)
Other, non-Hispanic		1.40 (0.78 (2.49)	1.39 (0.77, 2.50)
Hispanic		0.86 (0.62, 1.19)	0.82 (0.59, 1.14)
Income			
$100,000 or more		Ref	Ref
$60,000 to under $100,000		0.70 (0.53, 0.95)	0.70 (0.52, 0.93)
$30,000 to under $60,000		0.90 (0.66, 1.21)	0.85 (0.62, 1.16)
Less than $30,000		0.54 (0.37, 0.78)	0.52 (0.35, 0.76)
Metro			
No		Ref	Ref
Yes		1.24 (0.90, 1.71)	1.28 (0.93, 1.78)
Region			
Northeast		Ref	Ref
Midwest		1.14 (0.80, 1.64)	1.15 (0.80, 1.65)
South		1.29 (0.94, 1.76)	1.22 (0.88, 1.68)
West		1.04 (0.73, 1.48)	1.02 (0.72, 1.46)
Marital status			
Married or living with partner			Ref
Previously married			1.08 (0.79, 1.48)
Never married			0.90 (0.65, 1.25)
Importance of faith or spirituality			
Very important			Ref
Somewhat important			0.81 (0.61, 1.07)
Not too important			0.82 (0.58, 1.17)
Not important at all			0.69 (0.50, 0.97)

© 2025 Nouri S et al. *JAMA Network Open*.

Confidence in managing health problems			
I don't have any health problems			Ref
Not very confident			2.06 (1.13, 3.74)
Somewhat confident			1.37 (0.82, 2.30)
Very confident			2.06 (1.22, 3.48)

*Partially adjusted models were adjusted for age, gender, race/ethnicity, income, metro area, region.

^Fully adjusted models were adjusted for age, gender, race/ethnicity, income, metro area, region, marital status, importance of faith or spirituality, and self-efficacy.

© 2025 Nouri S et al. *JAMA Network Open*.

eTable 9. Odds of having documented medical wishes in writing, by self-reported serious illness.

Predictor	Odds ratio (95% CI); N = 1716		
	Unadjusted	Partially adjusted	Fully adjusted
Serious illness			
No	Ref	Ref	Ref
Yes	1.34 (1.04, 1.72)	1.26 (0.95, 1.68)	1.34 (0.99, 1.80)
Age			
18-44		Ref	Ref
45-64		2.04 (1.57, 2.67)	1.88 (1.42, 2.49)
65-74		5.75 (4.19, 7.89)	4.93 (3.51, 6.91)
75+		11.15 (7.12, 17.45)	9.59 (5.96, 15.43)
Gender			
Male		Ref	Ref
Female		1.34 (1.07, 1.68)	1.29 (1.02, 1.63)
Transgender or Non-Binary		4.66 (1.69, 12.87)	4.92 (1.75, 13.82)
Race/Ethnicity			
White, non-Hispanic		Ref	Ref
Black, non-Hispanic		0.61 (0.41, 0.90)	0.57 (0.37, 0.85)
Asian, non-Hispanic		1.03 (0.62, 1.70)	1.09 (0.66, 1.82)
Other, non-Hispanic		1.40 (0.79, 2.50)	1.43 (0.79, 2.57)
Hispanic		0.74 (0.52, 1.04)	0.71 (0.50, 1.01)
Income			
$100,000 or more		Ref	Ref
$60,000 to under $100,000		0.87 (0.65, 1.16)	0.86 (0.64, 1.15)
$30,000 to under $60,000		0.82 (0.60, 1.12)	0.81 (0.59, 1.12)
Less than $30,000		0.62 (0.43, 0.90)	0.64 (0.43, 0.94)
Metro			
No		Ref	Ref
Yes		1.07 (0.77, 1.49)	1.13 (0.81, 1.57)
Region			
Northeast		Ref	Ref
Midwest		0.97 (0.68, 1.40)	1.00 (0.69, 1.44)
South		1.07 (0.80, 1.47)	1.07 (0.77, 1.47)
West		0.96 (0.68, 1.36)	0.97 (0.68, 1.38)
Marital status			
Married or living with partner			Ref
Previously married			1.02 (0.74, 1.40)
Never married			0.88 (0.63, 1.24)
Importance of faith or spirituality			
Very important			Ref
Somewhat important			0.94 (0.70, 1.24)
Not too important			1.04 (0.73, 1.48)
Not important at all			0.72 (0.51, 1.01)

© 2025 Nouri S et al. *JAMA Network Open*.

Confidence in managing health problems			
I don't have any health problems			Ref
Not very confident			1.39 (0.73, 2.62)
Somewhat confident			1.49 (0.87, 2.53)
Very confident			2.21 (1.28, 3.79)

*Partially adjusted models were adjusted for age, gender, race/ethnicity, income, metro area, region.
^Fully adjusted models were adjusted for age, gender, race/ethnicity, income, metro area, region, marital status, importance of faith or spirituality, and self-efficacy.

© 2025 Nouri S et al. *JAMA Network Open*.

eTable 10. Frequency of ACP-related barriers and worries by self-reported serious illness.

	Overall, n (%)	Serious Illness, n (%)	No Serious Illness, n (%)	P-value
**Reasons for not having documented medical wishes in writing**	**N=1171**	**N=213**	**N=958**	
Haven't thought about it (e.g., too young, too healthy)	489 (43.1)	56 (29.8)	433 (45.9)	<.001
Surrogate who make decisions will know what they want	369 (32.2)	69 (36.8)	300 (31.3)	0.123
There are too many other things to worry about right now	357 (30.7)	84 (36.2)	273 (29.6)	0.059
Don't know how to begin or would need help to do it	306 (27.3)	72 (31.8)	234 (26.4)	0.113
Don't want to think about sickness and death	294 (24.7)	62 (26.7)	232 (24.3)	0.443
Don't think these documents will make any difference in their care	84 (6.8)	13 (5)	71 (7.2)	0.266
Have never heard of it	66 (6.5)	12 (5.5)	54 (6.7)	0.514
Want their doctors to make the decisions for them when needed	76 (5.7)	15 (6.4)	61 (5.6)	0.624
Don't have anyone that they can make responsible for decisions about their care	81 (5.6)	26 (8.8)	55 (5)	0.026
This is not something that people in their culture, religion, or family do	40 (5.4)	6 (5.2)	34 (5.4)	0.996
They are worried that having these documents will mean they get worse care	53 (5.1)	13 (5.8)	40 (5)	0.559
**Very worried about…**	**N=1803**	**N=361**	**N=1442**	
Not getting the best care because of their race, ethnicity, age, income, disability, or other reason	699 (36.9)	112 (26.6)	587 (39.3)	<.001
If they can't speak for themselves, their surrogates won't make the best-right decisions about their care	588 (31.9)	103 (29)	485 (32.6)	0.203
They won't understand how to make the best choices for treatment	406 (22.1)	77 (21.3)	329 (22.3)	0.724
They will have trouble managing all their appointments, tests, medications, and instructions for their doctor	355 (18.2)	55 (12.5)	300 (19.5)	0.003
Their surrogates will struggle caring for them during their illness and after their death (e.g., with finances or feelings)	296 (16.3)	42 (10.9)	254 (17.6)	0.002
They will not be able to continue living where they want to	279 (14.7)	46 (12.6)	233 (15.2)	0.22

© 2025 Nouri S et al. *JAMA Network Open*.

They may not have access to all the best treatment options	281 (14.7)	40 (11.9)	241 (15.4)	0.117
They will have trouble affording the medical care or support services they need	281 (14.6)	41 (9.8)	240 (15.8)	0.004
They will have a lot of pain, stress, anxiety, or depression	229 (11.7)	27 (5.5)	202 (13.1)	<0.001

© 2025 Nouri S et al. *JAMA Network Open*.

eTable 11. Engagement in ACP among people who are worried vs not worried. Grey = significantly higher rate of engagement among those who are worried vs not worried (P<0.05).

Worries	Any ACP Engagement, n (%)	Discussed surrogate with someone close to them, n (%)	Discussed medical wishes with someone close to them, n (%)	Discussed surrogate with clinician, n (%)	Discussed medical wishes with clinician, n (%)	Documented surrogate in writing, n (%)	Documented medical wishes in writing, n (%)
**Affording care (i.e., trouble affording medical care or support services)**							
Worried	815 (71.3)	812 (59.9)	814 (62)	810 (26.9)	811 (26)	784 (43.4)	781 (39.5)
Not Worried	976 (62)	971 (51.9)	971 (51.6)	969 (19.2)	973 (20.1)	931 (28.6)	925 (26.7)
**Surrogate decision-making (i.e., people close to them not making the best/right decisions about their care)**							
Worried	1360 (68.6)	1353 (58.6)	1357 (60.3)	1349 (23.9)	1355 (23.8)	1311 (38.1)	1306 (34.5)
Not Worried	435 (59.6)	433 (47.1)	433 (45)	434 (18.8)	433 (20)	408 (26.9)	404 (26.6)
**Access to best treatments (i.e., not having access to the best treatment options)**							
Worried	919 (70.6)	912 (59.9)	915 (61.1)	913 (27)	916 (27.2)	887 (39.2)	880 (36.5)
Not Worried	875 (61.9)	873 (51.1)	874 (51.5)	869 (18)	871 (18.2)	832 (31.1)	830 (28.2)
**High stress or symptom burden (i.e., having a lot of pain, stress, anxiety, or depression)**							
Worried	778 (69.2)	773 (59.9)	776 (60.7)	774 (26.6)	774 (27.6)	746 (41)	742 (38)
Not Worried	1015 (63.7)	1011 (52)	1011 (52.8)	1006 (19.4)	1012 (19.1)	969 (30.9)	964 (28.2)
**Caregiver burden (i.e., people close to them struggling to care for them during their illness and after their death)**							
Worried	827 (69.1)	822 (58.2)	825 (58.8)	821 (24.6)	823 (26.3)	792 (39.5%)	786 (36.3)
Not Worried	969 (63.3)	965 (53)	965 (54)	963 (20.5)	966 (19.4)	927 (31.4)	924 (29.2)
**Making medical decisions (i.e., understanding how to make the best choices for treatment)**							
Worried	1184 (67.8)	1178 (57.8)	1182 (60.3)	1175 (23.1)	1181 (24.6)	1133 (37.5)	1131 (33.7)
Not Worried	613 (63.6)	611 (51.7)	610 (49.3)	609 (21.7)	609 (19.8)	587 (31.2)	580 (30.1)
**Managing medical tasks (i.e., trouble managing their appointments, tests, medications, and doctors' instructions)**							
Worried	1137 (68.1)	1131 (59.1)	1135 (60.6)	1128 (22.7)	1134 (24.1)	1097 (36.6)	1091 (34.1)
Not Worried	659 (63.3)	656 (50.1)	656 (49.5)	655 (22.4)	655 (20.7)	622 (33)	619 (29.8)
**Discrimination (i.e., not getting the best care because of their race, ethnicity, age, income, disability, or other reason)**							
Worried	1304 (67.6)	1297 (57.4)	1299 (59)	1293 (22.5)	1298 (23.8)	1252 (36.5)	1252 (34)

© 2025 Nouri S et al. *JAMA Network Open*.

Not Worried	491 (62.8)	489 (50.9)	491 (49.6)	490 (23)	490 (20.1)	467 (32.1)	458 (28.8)
**Independence and function (i.e., not being able to continue living where they want to)**							
Worried	856 (66.7)	849 (57.7)	852 (58.6)	851 (26.1)	853 (25.3)	823 (36.6)	817 (33.1)
Not Worried	940 (65.4)	938 (53.2)	939 (53.8)	933 (19.4)	936 (20.2)	897 (33.6)	894 (31.4)

© 2025 Nouri S et al. *JAMA Network Open*.
