# Supplementary material for: Advance Care Planning Motivators Among Adults With Serious Illness
Source: JAMA Netw Open. 2025 Nov 4;8(11):e2541401. doi: 10.1001/jamanetworkopen.2025.41401 (PMC12587200; doi:10.1001/jamanetworkopen.2025.41401)
Supplement: Supplement 2. — Data Sharing Statement [file jamanetwopen-e2541401-s002.pdf]

Data Sharing Statement

Data
Data available: No

Additional Information
Explanation for why data not available: This patient-related information is not available for sharing. Upon request, a codebook can be made available to replicate analyses.
